# Levels of Anxiety, Depression, Self-Esteem, and Guilt in Women with High-Risk Pregnancies

**DOI:** 10.3390/jcm13237455

**Published:** 2024-12-07

**Authors:** Sevim Tuncer Can, Sevler Yildiz, Raziye Torun, Ibrahim Omeroglu, Hakan Golbasi

**Affiliations:** 1 Department of Perinatology, Izmir City Hospital, Izmir 35170, Turkey; drraziyetorun@gmail.com (R.T.); dribrahimomeroglu@gmail.com (I.O.); 2 Department of Psychiatry, Elazig Fethi Sekin City Hospital, Elazig 23280, Turkey

**Keywords:** high-risk pregnancy, anxiety, depression, self-esteem, guilt

## Abstract

**Objectives:** Pregnancy is an inherently delicate process characterized by physiological and psychological changes, even in the absence of any health complications. This study compares the levels of anxiety, depression, self-esteem, and guilt in women diagnosed with high-risk pregnancies to those in a control group consisting of women with healthy pregnancies. **Methods:** A total of 172 women participated in the study, 108 of whom had high-risk pregnancies, and 64 had healthy pregnancies. All participants were administered a semi-structured Sociodemographic Data Form, Beck Depression Inventory (BDI), Beck Anxiety Scale (BAI), Rosenberg Self-Esteem Scale (RSES), and Guilt Inventory (GI). The findings were statistically analyzed and compared. **Results:** Women with high-risk pregnancies had significantly higher scores on the BAI (*p* = 0.002), BDI (*p* = 0.035), and GI (*p* = 0.001) compared to the control group. In the logistic regression analysis for calculating the risk of high-risk pregnancy, the multivariate analysis revealed that living in rural areas posed 3.5 times higher risk for high-risk pregnancy compared to urban living (OR = 3.500, 95% CI = 1.484–8.254). Additionally, for every one-point increase in the GI score, the risk of high-risk pregnancy increased by 1.064 times (OR = 1.064, 95% CI = 1.017–1.114). In the patient group, significant positive correlations were found between the BAI score and BDI, RSES, and GI scores, while a significant negative correlation was observed between BAI and parity. There were also significant positive correlations between the BDI and RSES as well as the GI scores. Additionally, a positive significant correlation was found between the RSES and GI scores. **Conclusions:** Our findings may help in identifying the psychological states of women with high-risk pregnancies and

## 1. Introduction

Pregnancies that pose a risk to the mother and/or fetus due to pre-existing health conditions or complications that arise during pregnancy are classified as high-risk pregnancies and can develop due to both physiological and psychosocial factors [1]. Factors that increase risk include the mother’s age, pre-existing systemic diseases, as well as complications that develop during pregnancy such as premature rupture of membranes, placental abnormalities, intrauterine growth restriction, and preeclampsia [2,3]. It is estimated that approximately 10% of all pregnancies are classified as high-risk [4]. Research has shown that prenatal stress negatively affects not only the interpersonal relationships of pregnant women and their emotional bonding with the fetus but also the mother’s mental health during the postnatal period [5]. Furthermore, the increased levels of cortisol and catecholamines due to intense stress during high-risk pregnancies can increase the likelihood of pregnancy complications and negatively affect pregnancy outcomes [6]. The stress experienced by women during pregnancy can lead to miscarriage, preterm birth, and low birth weight cause emotional and behavioral health issues in the infant during the postnatal period [7,8]. Due to the potential threat to both the mother’s and fetus’s lives, the stress levels in high-risk pregnancies are higher compared to normal pregnancies [2]. The diagnosis of high-risk pregnancy, its associated stress, and its effects can negatively impact the coping abilities and psychological symptoms of the pregnant woman [9].

Many women experience psychological issues during pregnancy or postpartum [10]. The perinatal period is significant for maternal mental health conditions, including anxiety disorders, depression, bipolar disorder, and eating disorders [11]. It is believed that these mental health issues are more common in high-risk pregnancies compared to healthy pregnancies [12]. Higher levels of anxiety have been observed in women with high-risk pregnancies [13]. Smorti et al. reported that women with high-risk pregnancies had higher depression scores during the prenatal period [14].

Mental health disorders can affect an individual’s self-esteem and, in turn, trigger feelings of guilt [15]. Guilt is the feeling of remorse, conscience-stricken tension, and distress about a situation or action. These negative feelings often stem from cognitive errors related to a person’s perceived sense of responsibility [16]. Pregnant women naturally feel a sense of responsibility toward their unborn child [17]. Therefore, experiencing unwanted situations during pregnancy may impact the mother’s mental health and trigger feelings of guilt [18]. Learning a high-risk diagnosis during pregnancy, which is an important process for mother and baby, may affect the mental state of the expectant mother. For this reason, it is important to clarify the directions of psychosocial interventions that the pregnant woman may need after a high-risk diagnosis with the support of inexpensive and applicable scales. There is no study to date comparing high-risk pregnancies, which can lead to negative outcomes for both the fetus and the mother, with healthy pregnancies in terms of the psychological symptoms experienced by the expectant mother. Therefore, this study aimed to compare the psychological burden of high-risk pregnancies with healthy pregnancies.

## 2. Materials and Methods

This study was conducted following the ethical standards outlined in the 2013 revision of the Helsinki Declaration. Approval was obtained from the Tepecik Training and Research Hospital Non-Interventional Clinical Research Ethics Committee (approved on 10 October 2023 with approval number 2023/09-64). The study sample consisted of women diagnosed with high-risk pregnancies (*n* = 120) who presented to the Perinatology Outpatient Clinic of Tepecik Training and Research Hospital between November 2023 and February 2024. The control group included healthy pregnant women (*n* = 70) who visited the hospital for routine check-ups and had no known systemic or psychiatric diseases.

Voluntary participants from the high-risk pregnancy group were included based on the following inclusion criteria: being 18 years of age or older, being competent to answer the research questions, having no neurological or psychiatric disease diagnosis, receiving care for a high-risk pregnancy, and having a live fetus. High-risk pregnancies were defined as the presence of one or more of the following: preeclampsia, diabetes mellitus, vaginal bleeding, placenta previa, premature rupture of membranes, the threat of preterm labor, intrauterine growth retardation, fetal anomaly/distress, polyhydramnios/oligohydramnios, a multiples pregnancy, Rh incompatibility, pre-existing chronic diseases, and infections [19]. Healthy pregnant women from the control group were selected from those who visited the Obstetrics and Gynecology Department at Tepecik Training and Research Hospital for routine check-ups, with no known diseases or symptoms in the mother or the fetus.

Twelve participants from the high-risk pregnancy group and six from the healthy pregnancy group were excluded from the study due to non-compliance with the exclusion criteria. The scales were completed 1 month after the diagnosis of high-risk pregnancy. Data collection forms included self-reporting and were administered to the participants by the first author. Interviews lasted approximately 30 min for each participant. After obtaining written informed consent from all participants, the Sociodemographic Data Form, Beck Depression Inventory (BDI), Beck Anxiety Scale (BAI), Rosenberg Self-Esteem Scale (RSES), and Guilt Inventory (GI) were administered. In our study, validity and reliability studies of these scales were conducted in our country and their translated versions into Turkish were used.

### 2.1. Measures

Sociodemographic Data Form: a semi-structured form prepared by us that includes clinical data such as place of residence, duration of pregnancy, duration of illness, and presence of other comorbidities.

Beck Depression Inventory (BDI): measures the frequency of depression symptoms experienced by the individual. A high total score indicates a high level of depression experienced by the individual. It was developed by Beck et al. and a validity and reliability study was conducted in Turkey. The score range from the scale varies between 0–63. Moderate depression is indicated by 0–9 points, 10–16 points indicate mild depression, 17–29 points indicate moderate depression, and 30–63 points indicate severe depression. The cut-off score for Turkish society is accepted as 17. [20,21].

Beck Anxiety Scale (BAI): measures the frequency of anxiety symptoms experienced by the individual. A high total score indicates a high level of anxiety experienced by the individual. It was developed by Beck et al. and its validity and reliability study in our country was conducted by Ulusoy et al. The score range from the scale varies between 0–63. Minimal anxiety is indicated by 8–15 points, 16–25 points indicate mild anxiety, and 26–63 points indicate severe anxiety. The cut-off score for Turkish society is accepted as 17. [22,23].

Rosenberg Self-Esteem Scale (RSES): this scale was developed by Morris Rosenberg and is designed to measure self-esteem. Its validity and reliability study in our country was conducted by Cuhadaroglu et al. [24,25].

Guilt Inventory (GI): this was developed by Kugler and Jones; it has three subscales: trait guilt, situational guilt, and moral standards. It was adapted into Turkish by Altın. The situational guilt subscale was used in this study [26,27].

### 2.2. Statistical Analysis

The analyses were performed using SPSS (Statistical Package for the Social Sciences; SPSS Inc., Chicago, IL, USA) version 22. Descriptive statistics for categorical variables are presented as frequencies (*n*) and percentages (%), while continuous variables are expressed as mean ± standard deviation (Mean ± SD) and median with interquartile range (25th−75th percentile values). The comparison of categorical variables between groups was performed using the Pearson Chi-square test. The normality of continuous variables was assessed using the Kolmogorov–Smirnov test. For comparisons between two groups, the Student’s *t*-test was used for normally distributed variables, and the Mann–Whitney U test was used for non-normally distributed variables. For comparisons between more than two groups, the one-way ANOVA was applied for normally distributed variables, and the Kruskal–Wallis test was used for non-normally distributed variables. Spearman’s correlation test was employed to examine the relationship between continuous variables. Logistic regression analysis was used to calculate the risk of high-risk pregnancy. Significant variables from the binary comparisons were included in the multivariate model. Receiver operating characteristic (ROC) curves were plotted to evaluate the diagnostic value of scale scores in high-risk pregnancy diagnosis. A *p*-value of < 0.05 was considered statistically significant.

## 3. Results

A total of 172 participants were included in the study, with 108 women diagnosed with high-risk pregnancies and 64 women with healthy pregnancies. In the high-risk pregnancy group, the proportion of women living in rural areas (32.4%) was significantly higher compared to the control group (14.1%) (*p* = 0.008). Women in the high-risk pregnancy group had significantly higher scores on the Beck Anxiety Scale (BAI) (*p* = 0.002), Beck Depression Inventory (BDI) (*p* = 0.035), and Guilt Inventory (GI) (*p* = 0.001) compared to the healthy pregnancy group (Table 1).

In the logistic regression analysis conducted to calculate the risk of high-risk pregnancy, the multivariate analysis revealed that living in rural areas posed a 3.5 times higher risk for high-risk pregnancy compared to urban living (OR = 3.500, 95% CI = 1.484–8.254). Additionally, for each one-unit increase in the Guilt Inventory score, the risk of high-risk pregnancy increased by 1.064 times (OR = 1.064, 95% CI = 1.017–1.114) (Table 2).

**Figure 1:**
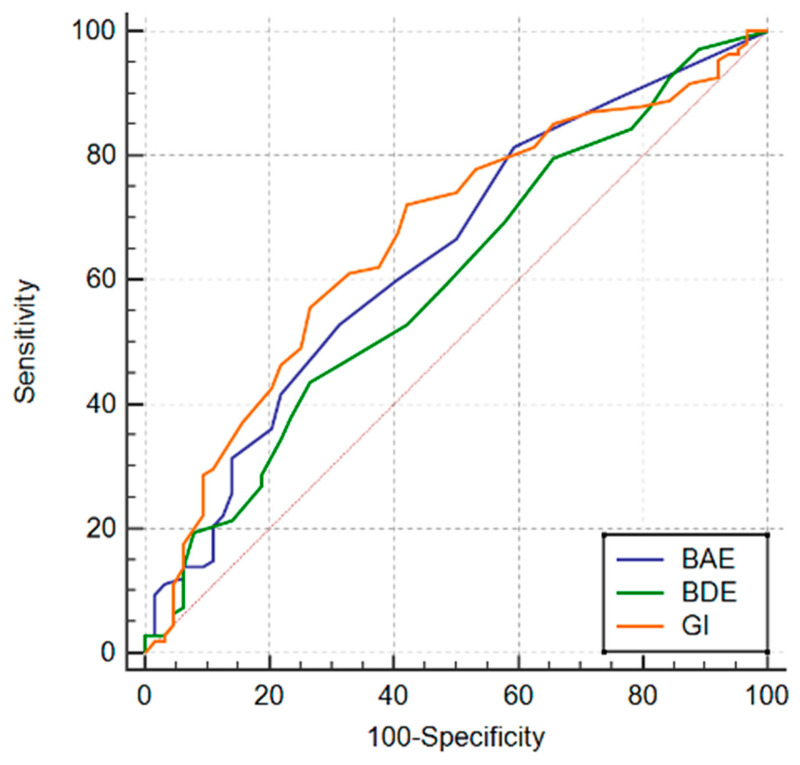
The ROC curve of BAI, BDI, and Guilt Inventory for risky pregnancies.

The ability of the Beck Anxiety Scale (BAS), Beck Depression Inventory (BDI), and Rosenberg Self-Esteem Scale (RSES) to predict high-risk pregnancy was evaluated through receiver operating characteristic (ROC) analysis, and their cut-off values were determined (Figure 1). For BAS, a cut-off value of 1 yielded a sensitivity of 81.5% and a specificity of 40.6%, indicating it as a good predictor. For BDI, a cut-off value of 9 yielded a sensitivity of 43.5% and a specificity of 73.4%, demonstrating it as a good predictor. For RSES, a cut-off value of 1.42 yielded a sensitivity of 90.7% and a specificity of 18.8%, suggesting it as a poor predictor. For the Guilt Inventory (GI), a cut-off value of 46 yielded a sensitivity of 72.2% and a specificity of 57.8%, indicating it as a good predictor (Table 3).

In the high-risk pregnancy group, there was a significant positive correlation between BAI scores and BDI, RSES, and GI scores, while a significant negative correlation was observed between BAI and parity. Additionally, significant positive correlations were found between BDI and RSES, as well as between BDI and GI scores. A positive significant correlation was also found between RSES and GI scores (Table 4).

When the etiologies of high-risk pregnancy in the patient group were analyzed, it was found that 13.9% had hypertension (HT), 21.3% had diabetes mellitus (DM), 12% had presentation anomalies, 12% had polyhydramnios, 5.6% had oligohydramnios, 13.9% had intrauterine growth restriction (IUGR), 4.6% had multiple pregnancies, 1.9% had antenatal bleeding, 2.8% had Rh incompatibility, 2.8% had acute surgical problems, and 9.3% had multiple risks. No significant differences were found between the etiologies in terms of BAI (*p* = 0.358), BDI (*p* = 0.540), RSES (*p* = 0.840), and GI (*p* = 0.769) scores (Table 5).

## 4. Discussion

In this study, where we compared high-risk pregnancies with healthy pregnancies in terms of psychological symptoms, we found that women in the high-risk pregnancy group had significantly higher levels of anxiety, depression, and guilt compared to the healthy pregnancy group. We observed that as the gestational week progressed, anxiety levels in the high-risk pregnancies decreased. Furthermore, anxiety and depression levels were found to be associated with a reduction in feelings of guilt and self-esteem. Independent of risk factors, as self-esteem decreased in high-risk pregnancies, feelings of guilt increased. Additionally, living in rural areas was found to pose a greater risk for high-risk pregnancy compared to urban living. In this study, we aimed to compare the psychological differences between high-risk and healthy pregnancies using developed scales. We observed that the Beck Anxiety Inventory (BAI), Beck Depression Inventory (BDI), and Guilt Inventory (GI) yielded different results in high-risk pregnancies compared to healthy pregnancies and that these scales may be useful in assessing the mental status of pregnant women.

The literature suggests that women experiencing or who have experienced high-risk pregnancies face a range of emotional issues, including fear, guilt, sadness, anxiety, and loneliness [2,28]. It is known that approximately 13% of pregnant women experience depression, and 21.7% experience anxiety [29]. High-risk pregnancies increase the risk of anxiety and depression [30]. Simmons and Goldberg reported that the label of “high-risk pregnancy” is associated with greater psychological distress [31]. Another study proposed that women are more likely to experience psychological distress when a health threat arises during pregnancy [32]. Saukko et al. reported that participants with high-risk pregnancies suffered from major depressive disorder [33]. Fairbrother et al. also found that high-risk pregnancies are associated with higher levels of anxiety during the perinatal period. In this study, pregnant women (*n*= 310) who scored at or above the cut-off point after completing postpartum screening measures for anxiety received a diagnostic interview for anxiety disorder (*n* = 115). The incidence of anxiety disorders in pregnancy was higher among women experiencing a medically moderate- or high-risk pregnancy compared with women experiencing a medically low-risk pregnancy. Pregnancies characterized by medical risks were associated with an increased likelihood of new-onset anxiety disorder [34]. Unlike our study, the severity of high-risk in these pregnant women was categorized. This study reported that the incidence of AD during pregnancy was higher among women with medically intermediate or high-risk pregnancies than among women with medically low-risk pregnancies.

Self-esteem serves as a protective factor against negative health behaviors in response to stressors [35]. During pregnancy, physical changes such as weight gain and stretch marks can affect a woman’s self-esteem [36]. However, personality traits and maternal feelings may help make these physical changes more acceptable and preserve self-esteem [37]. A study of 651 pregnant women found that as medical comorbidities increased, self-esteem decreased [38]. Scabia et al. reported that women with a previous adverse pregnancy outcome were more likely to be depressed, but this depression decreased postpartum, and that self-esteem remained high across all trimesters in high-risk pregnancies. This research was an observational longitudinal study. A pre-test (in the third trimester of pregnancy) and a follow-up measurement session (one month after birth) were applied. Self-efficacy and self-esteem levels were found to be unexpectedly high during and after pregnancy in all groups [39]. We evaluated pregnant women in the second trimester cross-sectionally. This difference may be due to the difference in pregnancy trimester. Even though she was diagnosed with a high-risk pregnancy, it may have increased the self-esteem of the pregnant woman close to birth in the 3rd trimester. In a study of 112 high-risk pregnant women, it was found that these women had lower self-esteem, as assessed by the Rosenberg Self-Esteem Scale [40]. Although this study did not have a control group, it is cross-sectional like our study.

As seen, self-esteem is related to levels of anxiety and depression. Our study similarly found that women in the high-risk pregnancy group had higher levels of anxiety, depression, and feelings of guilt compared to the healthy pregnancy group. Our findings support the results of many studies in the existing literature. While all pregnancies carry some level of risk, we observed that women diagnosed with high-risk pregnancies tend to experience more adverse psychological symptoms compared to those with healthy pregnancies.

Guilt is an emotion based on a sense of responsibility for harmful actions [16]. It has been noted that a mother’s feelings of guilt increase after experiencing a high-risk pregnancy [41]. Given that pregnancy inherently involves taking responsibility for the well-being of the fetus and the outcome of the pregnancy, it has been reported that expectant mothers often experience high levels of guilt [42]. While some women with high-risk pregnancies may blame the healthcare system [43], it is generally observed that the sense of guilt is directed towards themselves. In our study, independent of the cause of risk, we found that women with high-risk pregnancies experienced high levels of guilt. As guilt and self-esteem decreased, anxiety and depression levels increased. Isaacs et al. provided evidence that emotional experiences such as shock, fear, frustration, grief, isolation, loneliness, anger, sadness, and guilt are particularly pronounced during the high-risk pregnancy period [30]. “In this respect, our findings support most studies in the literature. Knowing the reasons for the feeling of guilt will shape the necessary support to be given to the pregnant woman in terms of maternal mental health”. Longitudinal studies on this subject will be able to clarify the reasons for feelings of guilt in high-risk pregnancies.

Living in rural areas is associated with an increased risk of high-risk pregnancy compared to urban living. Factors such as limited access to healthcare facilities, irregular gynecological check-ups, and poor socioeconomic conditions, which are more prevalent in rural areas, may contribute to the increased frequency of high-risk pregnancies. An analysis conducted in the United States found that pregnant individuals living in rural areas are at higher risk of intensive care unit (ICU) admissions and mortality compared to those living in urban areas [44,45]. In addition to the development level of the countries, access to health conditions will be beneficial for healthier follow-up of pregnancies.

In our findings, we observed that diabetes, hypertension, and intrauterine growth restriction were among the most common causes of high-risk pregnancy. In contrast, studies from the United Kingdom report hypertension, deep vein thrombosis, and postpartum hemorrhage as the leading causes [2]. These differences could be attributed to variations in national susceptibilities to metabolic diseases.

The limitations of our study include the relatively small sample size, cross-sectional design, differences in the week of diagnosis of high-risk pregnancy, differences in the severity of high-risk diagnosis in terms of fetal prognosis, use of self-report scales, and single-center design.

In conclusion, being aware of stressors in high-risk pregnancies and utilizing effective coping strategies could potentially improve the health outcomes for both the mother and the baby. Based on our findings, educating family members and healthcare professionals in hospitals where high-risk deliveries occur could enhance the psychosocial support provided to pregnant women, thereby improving their quality of life.

## Figures and Tables

**Table 1:** Comparison of all characteristics between groups.

		Patient (*n* = 108)		Control (*n* = 64)		*p*-Value
		*n*	%	*n*	%	
Age, Mean ± SD		30.06 ± 6.19		27.34 ± 4.56		0.071 *
Marital Status	Single	6	5.6	0	0	0.085 **
	Married	102	94.4	64	100.0	
Educational Level	Primary school or less	49	45.4	23	35.9	0.287 **
	High school	33	30.6	27	42.2	
	University	26	24.1	14	21.9	
Residence Location	Rural	35	32.4	9	14.1	0.008 **
	Urban	73	67.6	55	85.9	
Economic Status	Low	9	8.3	4	6.3	0.171 **
	Middle	85	78.7	57	89.1	
	High	14	13.0	3	4.7	
Employment Status	Employed	27	25.0	15	23.4	0.818 **
	Unemployed	81	75.0	49	76.6	
Smoking Status	Yes	20	18.5	7	10.9	0.186 **
	No	88	81.5	57	89.1	
Medical History	Yes	10	9.3	1	1.6	0.055 **
	No	98	90.7	63	98.4	
Family History	Yes	14	13.0	4	6.3	0.164 **
	No	94	87.0	60	93.8	
Gestasyonel Age	1st Trimester	2	1.9	0	0	0.758 **
	2nd Trimester	30	27.8	19	29.7	
	3rd Trimester	76	70.4	45	70.3	
Consanguinity	Yes	17	15.7	15	23.4	0.210 **
	No	91	84.3	49	76.6	
Gravida, median (IQR)		2.00 (1.00–3.00)		2.00 (1.00–3.00)		0.178 ***
Parity, median (IQR)		1.00 (0–2.00)		1.00 (0–1.50)		0.830 ***
Prenatal Care	Regular	106	98.1	64	100.0	0.530 **
	Irregular	2	1.9	0	0	
History of Anomaly in Previous Pregnancies	Yes	3	2.8	0	0	0.295 **
	No	105	97.2	64	100.0	
Mode of Conception	Natural	105	97.2	63	98.4	0.609 **
	Medication/IVF	3	2.8	1	1.6	
Week of Risk Detection	≤12th week	19	17.6	-		-
	13th–27th week	46	42.6			
	≥28th week	43	39.8			
BAI, median (IQR)		5.00 (2.00–10.00)		2.50 (0.50–6.00)		0.002 **
BDI, median (IQR)		8.00 (5.00–14.00)		6.00 (4.00–10.00)		0.035 ***
RSES, median (IQR)		0.75 (0.50–1.00)		0.75 (0.50–1.25)		0.886 ***
GI, Mean ± SD		50.93 ± 8.46		46.25 ± 8.41		0.001 *

* Student’s *t*-test, ** Chi-square test, *** Mann–Whitney U test were applied.

**Table 2:** Logistic Regression Analysis for Risky Pregnancy.

Variable	B	S.E.	*p*-Value	OR (95% Cl)
Residence Location (Ref: Urban)	1.253	0.438	0.004	3.500 (1.484–8.254)
BAI	0.049	0.035	0.153	1.051 (0.982–1.124)
BDI	–0.005	0.032	0.877	0.995 (0.935–1.060)
GI	0.062	0.023	0.007	1.064 (1.017–1.114)

B, beta coefficient; BAI, birth anxiety inventory; BDI, birth depression inventory; S.E., standard error.

**Table 3:** Specificity and sensitivity of measured parameters in identifying high-risk pregnancies.

Parameter	Area Under Curve (AUC)	*p*-Value	95% Confidence Interval (CI)		Sensitivity (%)	Specificity (%)	Positive Predictive Value (PPV) (%)	Negative Predictive Value (NPV) (%)
			Lower Limit	Upper Limit				
BAI > 1	0.642	0.001	0.565	0.713	81.5	40.6	69.8	56.5
BDI > 9	0.596	0.032	0.519	0.670	43.5	73.4	73.4	43.5
RSES ≤ 1.42	0.506	0.891	0.429	0.583	90.7	18.8	65.3	54.5
GI > 46	0.668	<0.001	0.592	0.738	72.2	57.8	74.3	55.2

BAI, Beck Anxiety Inventory; BDI, Beck Depression Inventory; GI, Guilt Inventory; RSES, Rosenberg Self-Esteem Scale.

**Table 4:** Correlation of scales in the patient group.

		BAI	BDI	RSES	GI
BDI	r	0.539			
	*p*	<0.001			
RSES	r	0.519	0.428		
	*p*	<0.001	<0.001		
GI	r	0.415	0.433	0.398	
	*p*	<0.001	<0.001	<0.001	
Age	r	−0.044	−0.022	−0.085	0.016
	*p*	0.651	0.818	0.382	0.869
Gravida	r	−0.152	0.062	0.028	−0.081
	*p*	0.117	0.527	0.771	0.407
Parite	r	−0.227	0.045	−0.066	−0.084
	*p*	0.018	0.641	0.497	0.389
Week of Risk Detection	r	0.005	−0.126	−0.105	0.008
	*p*	0.959	0.195	0.280	0.937

BAI, Beck Anxiety Scale; BDI, Beck Depression Inventory; GI, Guilt Inventory; RSES, Rosenberg Self-Esteem Scale.

**Table 5:** Comparison of scale scores according to the etiology of high-risk pregnancy.

	*n* (%)	BAI	BDI	RSES	GI
Hypertension	15 (13.9)	6.00 (2.00–20.00)	8.00 (4.00–16.00)	0.66 (0.25–1.00)	48.6 ± 8.0
Diabetes	23 (21.3)	4.00 (2.00–9.00)	8.00 (5.00–16.00)	0.75 (0.50–1.25)	52.0 ± 8.3
Presentation anomaly	13 (12.0)	7.00 (5.00–12.00)	11.00 (7.00–16.00)	0.84 (0.50–1.25)	52.5 ± 9.5
Polyhydramnios	13 (12.0)	4.00 (1.00–9.00)	9.00 (4.00–12.00)	0.75 (0.25–0.75)	50.8 ± 7.7
Oligohydramnios	6 (5.6)	6.50 (4.00–12.00)	9.00 (2.00–20.00)	0.75 (0.50–0.83)	46.7 ± 12.3
IUGR	15 (13.9)	7.00 (3.00–16.00)	9.00 (5.00–14.00)	0.75 (0.50–1.25)	53.6 ± 6.8
Multiple pregnancy	5 (4.6)	2.00 (2.00–3.00)	5.00 (5.00–5.00)	0.50 (0.50–0.50)	49.4 ± 4.8
Antepartum hemorrhage	2 (1.9)	1.50 (0.00–3.00)	4.50 (1.00–8.00)	0.67 (0.25–1.08)	53.0 ± 2.8
Rh incompatibility	3 (2.8)	11.00 (0.00–12.00)	8.00 (4.00–13.00)	1.50 (0.25–1.75)	49.0 ± 5.6
Acute surgical problem	3 (2.8)	6.00 (4.00–31,00)	8.00 (5.00–14.00)	0.50 (0.25–1.75)	54.3 ± 6.4
Multiple risks	10 (9.3)	3.50 (2.00–10.00)	6.00 (5.00–10.00)	0.75 (0.50–1.00)	48.5 ± 12.1
*p*		0.358 *	0.540 *	0.840 *	0.769 **

* Kruskal–Wallis analysis, ** one-way ANOVA analyses were applied. BAI, Beck Anxiety Scale; BDI, Beck Depression Inventory; DM, Diabetes Mellitus; GI, Guilt Inventory; HT, Hypertension; IUGR, Intrauterine Growth Restriction; RSES, Rosenberg Self-Esteem Scale.

## Data Availability

Data is not available due to ethical reasons. Further inquiries can be directed to the corresponding author.
